# Prospective clinical study testing the efficacy and safety of a new formula to increase the precision of oxygen therapy in the initiation phase of cardiopulmonary bypass

**DOI:** 10.1177/02676591221100743

**Published:** 2022-05-24

**Authors:** Jan Turra, David Riesterer, Christoph Eisner, Folker Wenzel, Andreas Möbius, Matthias Karck, Rawa Arif, Christoph Lichtenstern, Dania Fischer

**Affiliations:** 1 Department of Cardiothoracic Surgery, 54278 University Hospital Heidelberg, Heidelberg, Germany; 2 Department of Anesthesiology, 27178 University Hospital Heidelberg, Heidelberg, Germany; 3 Furtwangen University, 120264 University Schwenningen, Schwenningen, Germany

**Keywords:** Cardiac surgery, heart-lung-machine, formula, inspiratory oxygen fraction, arterial oxygen partial pressure, gas blender, setting

## Abstract

**Introduction:**

During cardiopulmonary bypass (CPB), supranormal concentrations of oxygen are routinely administered with the intention to prevent cellular hypoxia. However, hyperoxemia may have adverse effects on patient outcome. Oxygen settings are based on the perfusionist’s individual work experience rather than profound recommendations and studies analyzing the effect of oxygen levels are in need of methodological improvement. We aimed to advance perfusion technique by developing and clinically applying a formula for tailored oxygen therapy in CPB.

**Methods:**

A formula to precalculate the oxygenator setting before CPB was developed. The newly-derived formula was then evaluated in a prospective, single-center pilot study to test whether a predefined arterial partial oxygen pressure (PaO2) of 150–250 mmHg could be reached. 80 patients were enrolled in the study between April and September 2021.

**Results:**

The mean oxygen fraction calculated for the setting of the gas blender was 52% ±0,12. The mean PaO2 after initiation of the CPB was 193 ± 99 mmHg (min-max: 61-484, median 163 mmHg). 38.75% of the values were in the desired PaO2 corridor of 150 to 250 mmHg. 8.75% of all PaO2 values were below <79.9 mmHg, 31.25% between 80 and 149.9 mmHg, 38.75% between 150 and 249.9 mmHg and 21.25%>250 mmHg.

**Conclusions:**

Conceptually, perfusion technique should be goal-directed, guided by objective parameters and formulas. Although the optimal CPB oxygenation target remains unknown, it is nevertheless important to develop strategies to tailor oxygen therapy to aid in creating evidence as to what level of oxygen is best for patients during CPB. The formula we derived needs further adjustments to increase results in the target range.

## Introduction

The use of the heart-lung machine (HLM) for temporary extracorporeal organ replacement enables complex surgical treatment of organs inside the thoracic cavity — the heart, lungs, and other pleural or mediastinal structures. The cardiovascular perfusionist individually adjusts and operates the HLM, ensuring that blood circulation and gas exchange meet the patient’s metabolic needs. Especially the adequate supply of oxygen (O_2_) through the HLM perfusion during cardiopulmonary bypass (CPB) is essential to enable mitochondrial oxidative phosphorylation, ensuring energy supply. However, it does not necessarily follow that ‘too much’ oxygen is the best solution to ‘not enough’. While the harms of profound hypoxia are well recognized, the effects of hyperoxia, that is, supraphysiological arterial partial pressure of O_2_ (P_a_O_2_), are less clear. Clinical studies from a variety of settings demonstrate that perioperative hyperoxia may be associated with adverse events such as higher levels of cardiovascular complications.^1–3^ Greater attention should be paid to the precision with which oxygen is administered, presumably especially in patients with acute myocardial infarction as the effect of hyperoxia may be especially profound in this cohort. For example, Stub et al. found that supplemental oxygen therapy in patients with ST-elevation-myocardial infarction but without hypoxia may increase early myocardial injury and was associated with larger myocardial infarct size.^4^ The adverse effects of hyperoxia may be caused by increased oxidative stress through increased generation of reactive oxygen species (ROS), which are considerably more reactive than their corresponding non-radical form.^5^ They are generated within mitochondria during normal cellular metabolism, but ROS generation is accelerated under certain conditions including hyperoxia.^6^ Furthermore, hyperoxia has a vasoconstrictive effect and may result in perfusion heterogeneity and tissue injury.^7–9^ Previous studies in patients undergoing coronary artery bypass surgery (CABG) suggest reduced myocardial damage when avoiding extreme perioperative hyperoxia (>400 mmHg). P_a_O_2_ levels of more than 200 to 300 mmHg during CPB are not exceptional.^10–15^ But other studies failed to show that avoidance of mild hyperoxia compared to moderate hyperoxia, a near-physiological oxygen strategy reduced myocardial damage in patients undergoing CABG.^16^ Despite the fact that current evidence seems insufficient to determine optimal oxygen targets, it is important to develop a formula that helps to reach a predefined target. This might support the design and conduction of future clinical studies.

In the preparatory phase before the initiation of the CPB, it is difficult for the perfusionist to anticipate the optimal HLM settings to guarantee sufficient arterial oxygenation without risking hyperoxia. The cumulative arterial oxygenation that develops in the first couple of minutes after initiation of the CPB depends on many factors on the patient side such as body surface area, cardiac output, oxygen consumption and total blood volume as well as factors on the HLM side such as the priming volume, calculated pump flow and the oxygen transfer capacity of the oxygenator.

The perfusionist’s technique to adjust the settings of the HLM and to initiate the CPB depends heavily on individual work experience rather than profound recommendations. There seems to be a wide range of practice in relation to the optimum oxygen setting during CPB.^14^ Neither the currently available literature, nor the manuals of HLM and disposable sets, provide a scientifically founded recommendation nor a gold standard regarding the individualized HLM setting of the inspiratory oxygen fraction (F_i_O_2_).

Although the optimum target range for P_a_O_2_ has not been established in clinical trials yet, the development of a precise formula to target oxygenation is valuable as it may help to design and conduct future studies for gaining this missing evidence.

In this study, we developed and clinically tested a formula to set a specific preoperative F_i_O_2_ on the gas blender for the individual patient, leading to a desired target mean value of 200 ± 50 mmHg of the P_a_O_2_ resulting after initiation of CPB. Our objective was to move closer to a goal-directed perfusion, customizing patient care by taking into account their size, hemoglobin, and individual physiologic requirements.

## Methods

### Derivation of the formula for calculating the gas blender’s F_i_O_2_

The formula was derived as will be explained in the results section. It is based on standard formulas for the calculation of body surface area (BSA), cardiac output (CO), total blood volume (V_B_), oxygen consumption (VO_2_) as well as HLM output and information on the oxygen transfer of the oxygenator, all of which will be detailed in the results section.

### Ethics

The study protocol was approved by the Institutional Ethics Committee of the Heidelberg University (reference number: S-088/2021). The study was registered prior to patient enrollment at the German Clinical Trial Register (DRKS00025063). https://www.drks.de/drks_web/navigate.do?navigationId=trial.HTML&TRIAL_ID=DRKS00025063

### Study design and setting

This prospective, single-center pilot study to test the derived formula was conducted by the Department of Cardiothoracic Surgery and the Department of Anesthesiology of the Heidelberg University Hospital, Germany between April and September 2021. A sample size calculation ahead of the study was not possible as this is a pilot study. We assumed that veritable results could be achieved with a cohort of 80 patients.

### Participants

Consecutive patients scheduled for elective cardiac surgery at our institution were assessed for eligibility and written informed consent was obtained on the day before surgery. The inclusion criteria were age ≥18 years, elective cardiac surgery with CPB, hemoglobin >9 g/dL, hematocrit >30% and informed consent. Exclusion criterion was the need of a preoperative cardiac support system.

### Study protocol anesthesia and surgery

The treating anesthesiologist determined the ventilation and fluid management before extracorporeal circulation without a study-related predefined goal-directed therapy algorithm. An inhalational agent (sevofluran) combined with sufentanil were used for anesthesia maintenance. The only deviation from the standard procedure was the use of the new formula for the adjustment of the heart-lung machine by the perfusionist. All patients received a midline sternotomy and a variety of cannulations in preparation for CPB. The return cannula was connected to the arterial line (3/8 × 3/32-inch tubing) and the drainage cannula was connected to the venous line (1/2 × 3/32-inch tubing) of the HLM.

### Study protocol heart-lung-machine

We decided to set our P_a_O_2_ target range at 150–250 mmHg, as this is the range that is described as *“usual practice”* in important clinical trials on the effect of hyperoxia in CPB.^17^ Compared to physiological P_a_O_2_ at sea level of 75–100 mmHg,^18^ there is a margin of safety included as we did not want to risk states of hypoxia.

The set-up of our HLM was as followed: we used a LivaNova S5 (LivaNova PLC, London, United Kingdom) heart-lung machine as well as a Medtronic Fusion^®^ (Medtronic, Dublin, Ireland) oxygenator module with integrated arterial filter and open hard-shell venous reservoir. The total volume of the venous reservoir was five liters and the maximum flow through the microporous hollow fiber oxygenator was 7.0 L/min. The arterial roller pump was checked for occlusion to ensure that the calculated blood flow could be achieved. The priming of the HLM was carried out with 1000 mL of balanced electrolyte solution including heparin at 10 IU/mL. Before initiation of extracorporeal circulation, the priming solution was preheated to the patient’s body temperature. The F_i_O_2_ was set after calculation with the newly developed formula for every individual patient and the gas flow was set at 2.0 L/min. After setting all parameters, CPB was started. After the full pre-calculated cardiac output (100% flow) was reached, the ventilation was disconnected by the anesthesiologist and the two-minute equilibration time began. After the two minutes, a venous and arterial blood gas analysis was taken from the blood collection port of the HLM. After collecting the gas samples, the surgeon and anesthesiologist carried on with the procedure as planned.

### Measurements

For the arterial blood gas (ABG) analysis, the RAPIDPoint 500 System^®^ from Siemens Healthineers (Munich, Germany) was used to measure patients’ PaO2 at the designated time points in the operating room. The principle of measurement based on the electrochemistry analytes with potentiometry, amperometry and conductimetric methods to convert the potential generated by the sensor to an electrical signal.^18^

### Primary outcome

The primary outcome was the rate of P_a_O_2_ in the target range of 150 to 250 mmHg.

### Secondary outcomes

Rate of P_a_O_2_ < 79.9 mmHg or >250 mmHg, precalculated values of CO, V_B_, oxygen consumption VO_2_ compared to actual values after initiation of CPB.

### Statistical methods

Data was collected with the aid of an electronic database system (Microsoft Excel®, Microsoft Deutschland GmbH, Unterschleißheim). PASW Statistics v18 Multilingual (IBM® Deutschland GmbH, Ehningen) was used for statistical analyses. Descriptive statistics were used to describe the patient sample and data set. Results are presented as n (%), mean (SDs), or median (interquartile range).

## Results

### Formula development

The formula developed in this study is based on standard formulas for the calculation of BSA, CO, V_B_, VO_2_ as well as HLM output and information on the oxygen transfer of the oxygenator, all of which will be detailed here. Generally, a cardiac index (CI) of 2.5 L/min/m^2^ was assumed.

Formulas can be found in Table 1, they are assorted according to patient- and HLM-specific formulas as well as formulas that allow to calculate expected values once a patient is connected to the HLM (+).

**Table 1.** Formulas on which the derivation is based HLM = heart-lung-machine; + = values once a patient is connected to the HLM.

	Variable	Formula	Abbreviations
Patient	Body surface area (BSA)^19^	BSA=height×weight÷3600	• *BSA* = body surface area (m^2^)
			• ℎ𝑒𝑖𝑔ℎ𝑡 = body hight (cm)
			• 𝑤𝑒𝑖𝑔ℎ𝑡 = body weight (kg)
	Cardiac output (CO)^20^	CO=CI×BSA	• *CO* = cardiac output (mL/min)
			• *CI* = Cardiac index (l/min/m²)
			• *BSA* = body surface area (m^2^)
	Total blood volume (V_B_)^21^	$V_{B}=BW\times V_{Pat}$	• V_B_ = total patient blood volume (ml)
			• BW = patient body weight (kg)
			• VPat = blood volume/kg/BW
	Oxygen consumption (VO_2_)^22,23^	$VO_{2}=(\left(\left(S_{a}O_{2}-S_{v}O_{2}\times \left(Hct\mathrm{÷3}\right)\right)\times \text{CO}\right)\times 0,134$	• $VO_{2}$ = Oxygen consumption (mL O_2_/min)
			• $S_{a}O_{2}$ = arterial hemoglobin oxygenation (%)
			• $S_{v}O_{2}$ = venous hemoglobin oxygenation (%)
			• $Hct_{i}$ = initial hematocrit (g/dL)
			• HZV = Herz-Zeit-Volumen (mL/min)
+	Expected hemoglobin (Hb_exp_) after initiation of the cardiopulmonary bypass^24^	$Hb_{\exp}=\left(\frac{\left(Hb_{1}\times V_{B}\right)}{\left(\left(V_{B}+V_{P}\right)-RAP\right)}\right)$	• $Hb_{\exp}$ = expected hemoglobin (g/dL)
			• $Hb_{1}$ = initial hemoglobin (g/dL)
			• $V_{B}$ = total patient blood volume (mL)
			• $V_{P}$ = HLM priming volume (mL)
			• RAP = Retrograde autologous priming (mL)
	Expected venous hemoglobin oxygenation (S_v_O_2exp_)^22,23^	$S_{v}O_{2\exp}=S_{a}O_{2}-\left({\text{VO}}_{2}÷\left(\left(0\mathrm{.134*H}b_{\exp}\right)\times \text{CO}\right)\right)$	• $S_{v}O_{2\exp}$ = expected venous hemoglobin saturation (%)
			• $S_{a}O_{2}$ = arterial hemoglobin oxygenation (%)
			• $VO_{2}$ = oxygen consumption (mL O_2_/min)
			• $Hb_{\exp}$ = expected hemoglobin (g/dL)
			• *CO* = cardiac output (mL/min)
	Expected oxygen consumption after initiation of the cardiopulmonary bypass^22,23^	$VO_{2\exp}=\left(S_{a}O_{2}-S_{v}O_{2\exp}\right)\times \left(Hb_{\exp}\times \text{CO}\right)\mathrm{\times 0}\mathrm{.134}$	• $VO_{2\exp}$ = expected oxygen consumption (ml O_2_/min)
			• $S_{a}O_{2}$ = arterial hemoglobin oxygenation (%)
			• $S_{v}O_{2\exp}$ = expected venous hemoglobin oxygenation (%)
			• $Hb_{\exp}$ = expected hemoglobin (g/dL)
			• *CO* = cardiac output (mL/min)
	Roller arterial pump (= HLM-) output^23^	$Q_{b+}=\pi \times {\text{r}}^{2}\times l\times \text{rpm}$	• $Q_{b}$ = HLM-output
HLM			• $r^{2}$ = Inner diameter of the pump segment
			• 𝑙 = contact length of roller pump and tube
			• *rpm* = rotations per minute (s)
	Oxygen addition $\left(O_{2+}\right)$ ^25^	$O_{\mathrm{2+}}=(\left({\text{P}}_{\text{a}}O_{2}-150\right)\mathrm{\times 0}\mathrm{.0031}(\left(\text{Priming}+{\text{V}}_{\text{B}}\right)÷10$	• $O_{2+}$ = additional oxygen (mL/dL)
			• $P_{a}O_{2}$ = arterial hemoglobin oxygenation (%)
			• *Priming* = priming volume of the HLM (mL)
			• $V_{B}$ = total patient blood volume (mL)
	Oxygen addition via CO $\left(O_{2+}+CO\right)$ ^25^	$O_{\mathrm{2+}\text{CO}}=(\left({\text{P}}_{\text{a}}O_{2}-150\right)\mathrm{\times 0}\mathrm{.0031}(\left(\text{Priming}+{\text{V}}_{\text{B}}\right)÷$	• $O_{2+co}$ = additional oxygen CO (mL/dL)
			• $P_{a}O_{2}$ = arterial hemoglobin oxygenation (%)
			• *Priming* = priming volume of the HLM (mL)
			• $V_{B}$ = total patient blood volume (mL)
			• *CO* = cardiac output (mL/min) (%)

From these established formulas, we moved on to develop a formula to calculate the oxygen that is added through the oxygenator (Table 2), which additionally takes into account both the priming volume of the HLM and the volume output of the HLM.

**Table 2.** Formulas to calculate the additional oxygen added by the oxygenator.

Variable	Formula	
Oxygen addition $\left(O_{2+}\right)$ ^25^	${\text{O}}_{2+}=\left(\left(\left({\text{P}}_{\text{a}}{\text{O}}_{2}-150\right)\text{×0}\text{.}0031\right)\text{×}\left(\left({\text{Priming}+\text{ V}}_{\text{B}}\right)\text{÷}100\right)\right)$	• $O_{2+}$ = additional oxygen (mL/dL)
		• $P_{a}O_{2}$ = partial pressure of oxygen (mmHg)
		• *Priming* = priming volume of the HLM (mL)
		• $V_{B}$ = total patient blood volume (mL)
Oxygen addition via CO $\left(O_{2+CO}\right)$ ^25^	${\text{O}}_{2+\text{CO}}=\left(\left(\left({\text{P}}_{\text{a}}{\text{O}}_{2}-150\right)\text{×0}\text{.}0031\right)\text{×}\left(\left({\text{Priming}+\text{ V}}_{\text{B}}\right)\text{÷}1000\right)\right)\text{x}\left(\text{CO x }10\right)$	• $O_{2\;+\;CO}$ = additional oxygen CO (mL/dL)
		• $P_{a}O_{2}$ = partial pressure of oxygen (mmHg)
		• *Priming* = priming volume of the HLM (mL)
		• $V_{B}$ = total patient blood volume (mL)
		• CO = cardiac output (mL/min)

Furthermore, we added the oxygen transfer capacity of the Medtronic Fusion oxygenator. The trendline is y = 462.03x–43.025.^25^

Based on this, we developed a formula (Table 3) for calculating the gas blender’s setting of the F_i_O_2_, which translates into the final formula, which was then used in our clinical study (Table 3).

**Table 3.** A+B: Formula for calculating the gas blender’s setting of the oxygen fraction.

Variable	Formula
A ${\text{F}}_{\text{i}}{\text{O}}_{2\;}=\left(\left(\left(\left(\left(\left({\text{P}}_{\text{a}}{\text{O}}_{2}-150\right)\text{ ×0}\text{.}0031\right)\text{×}\left(\left({\text{Priming}+\text{ V}}_{\text{B}}\right)\text{÷}1000\right)\right)\text{×}\left(\text{CO ×}10\right)\right)+43\text{.}024\right){+\text{ VO}}_{2\text{exp}}\right)\text{÷}462$	• $F_{i}O_{2\;}$ = $O_{2\;}$ fraction in the gas flow (%)
	• $P_{a}O_{2}$ = partial pressure of oxygen (mmHg)
	• *Priming* = priming volume of the HLM (mL)
	• $V_{B}$ = total patient blood volume (mL)
	• CO = cardiac output (mL)
	• $VO_{2\text{exp}}$ = expected oxygen consumption (mL O2/min)
B ${\text{F}}_{\text{i}}{\text{O}}_{2}=\;\frac{{\text{V}}_{+}{\text{O}}_{2}+\left[{\text{PaO}}_{2}\text{*0}{\text{.}0031\text{*V}}_{\text{B}}\text{ ÷ }100\right]+\;43\text{,}02}{462\text{.}03}$	• $F_{i}O_{2\;}$ = O2 fraction in the gas flow (%)
	•V + O2- = volume of additional O2
	• $P_{a}O_{2}$ = partial pressure of oxygen (mmHg)
	•*Priming* = priming volume of the HLM (mL)
	• $V_{B}$ = total patient blood volume (mL)
	• CO = cardiac output (mL)

### Clinical study

The study enrolled 80 consecutive patients according to the availability of the study team. The mean hemoglobin level (standard deviation, SD) just prior to the initiation of the CPB was 11.77 g/dL (±1.39). Table 4 shows further baseline characteristics of the patients.

**Table 4.** Patient baseline characteristics.

Patient characteristics	*n* = 80	SD
Gender
Male	63 (78.75%)	
Female	17 (21.25%)	
Hemoglobin	11.77 g/dl	±1.39
Hematocrit	34.6%	±4.06
Height (cm)	173.11	±8.19
Body weight (kg)	86.26	±19.45
BSA (m^2^)	2.04	±1.36
BMI (kg/m^2^)	29,23	±5,91
CO (l/min)	5.1	±0.52
Chronic obstructive pulmonary disease	6 (7.5%)
Hypertension	52 (65%)	
Diabetes type II	23 (28.75%)	
Carotid artery stenosis	6 (7.5%)
Left ventricular function
Normal	56 (70%)	
Slightly reduced	12 (15%)	
Moderately reduced	6 (7.5%)	
Highly reduced	6 (7.5%)	
Type of operation
Coronary artery bypass surgery	34	
AVR	9	
MVR	11	
CABG + AVR	12	
AVR + MVR	4	
Other	10	

After application of our formula in the preparatory phase of CPB, the mean oxygen fraction we calculated for the setting of the gas blender (F_i_O_2_) was 52% (±0.12). The mean P_a_O_2_ before initiation of the CPB was 214.67 ± 96.33 mmHg.

**Figure 1.**
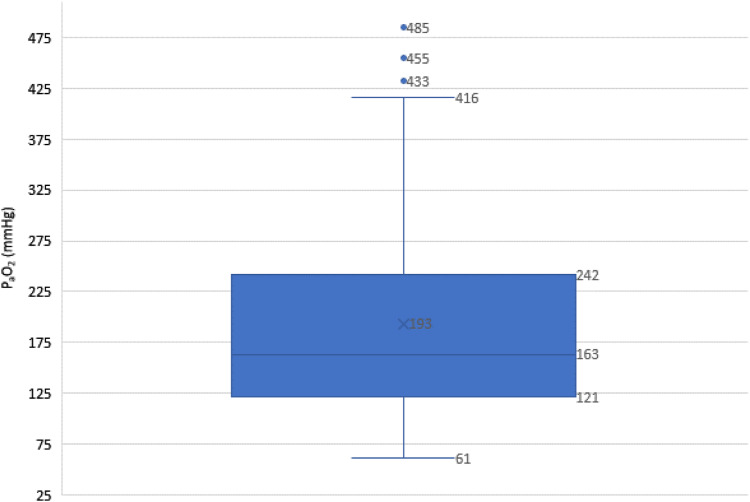
Box plot showing the distribution of arterial oxygen partial pressures, including median, upper and lower quartiles, *n* = 80. Outliers that differ significantly from the rest of the dataset are plotted as individual points.

The mean arterial P_a_O_2_ after initiation of the CPB was 193 ± 99 mmHg (min-max: 61–484, median 163 mmHg). 38.75% of the values were in the desired P_a_O_2_ corridor of 150 to 250 mmHg. 8.75% of all P_a_O_2_ values were below <79.9 mmHg, 31.25% between 80 and 149.9 mmHg, 38.75% between 150 and 249.9 mmHg and 21.25% > 250 mmHg. Figure 1

Our final formula was based on different pre-assumptions or pre-calculations, which are shown in Table 5, contrasted with the values that were calculated or measured after initiation of CPB.

**Table 5.** Expected values versus measured values.

Variable	Pre-assumed/pre-calculated value			Value calculated/measured post-CPB-initiation	
Cardiac output (CO) in l/min/m^2^ Formula: ${\text{CO }=\text{ VO}}_{2\;}\text{÷}\left(10\text{×BSA}\text{×}\left({\text{S}}_{\text{a}}{\text{O}}_{2\text{ prä HLM}}{-\text{ S}}_{\text{v}}{\text{O}}_{2\text{ prä HLM}}\right)\right)\;$ ^22,23^	2.5			Resulting P_a_O_2_ < 79,9 mmHg	3.5
				Resulting P_a_O_2_ 80–149.9 mmHg	3.63
				Resulting P_a_O_2_ 80–150–249.9 mmHg	2.4
				Resulting P_a_O_2_ > 250 mmHg	1.95
Total blood volume (V_B_)^21^	Mosteller	Male	6.99 ± 1.26	Total male = 5.79 ± 2.28 Total female = 4.15 ± 1.3	
		Female	5.95 ± 1.27		
	Nadler	Male	5.41 ± 0.64		
		Female	4.62 ± 0.72		
Oxygen consumption (VO_2_) in ml O_2_/min^22,23^	159.70 ± 55.35 mL O_2_/min			155.56 ± 42 mL O_2_/min	
Hemoglobin (Hb_exp_) in g/dL after initiation of the CPB^24^	10.01 ± 1.28 g/dL			9.85 ± 1.38 g/dL	
Hematocrit (Hb_exp_) in % after initiation of the CPB^24^	30.03 ± 3.83%			29 ± 4.02%	
Oxygen consumption after initiation of the CPB	200 mmHg			193 ± 99 mmHg	

CPB = cardiopulmonary bypass.

## Discussion

Tissue health depends on adequacy of perfusion and oxygenation, which should ideally be tailored to the needs of each individual patient. One of the challenges facing clinical perfusionists is to anticipate these individual oxygen needs and adjust HLM settings accordingly. The present study addresses this problem and describes the development of a formula to aid the perfusionist in targeting a pre-defined arterial oxygenation. To our knowledge, it is the first explorative evaluation of a formula for the objective setting of the gas blender before initiation of the CPB.

Conceptually, goal-directed perfusion technique should be guided by objective parameters and formulas. The P_a_O_2_ target range in our study was set at 150 to 250 mmHg as this corresponds to the standard of care used in clinical perfusion studies.^17^ However, it needs to be kept in mind that there are currently no definitive studies showing which range is best for patients in the initiating phase of CPB. In cardiac surgery, patients are frequently treated with higher concentrations of oxygen to guard against myocardial and cerebral hypoxia, although physiologically, P_a_O_2_ is between 75 and 100 mmHg at sea level.^18^ It is important to determine whether an initially high P_a_O_2_ is truly pernicious for cardiac patients. It would be a feasibly option to prospectively compare patient groups with different P_a_O_2_-target ranges by biochemically analyzing the production of reactive oxygen species (ROS) and organ damage markers. The groups could ideally be separated by application of a more advanced version of our formula.However, especially in settings of myocardial ischemia-reperfusion, targeting a predefined arterial oxygenation and avoiding hyperoxia is important as evidence suggests detrimental effects of hyperoxia compared to normoxia or hypoxia.^4,26,27^ The pathophysiology involves numerous injurious pathways including oxidative stress and formation of reactive iron species. Nevertheless, until now, avoiding modest hyperoxemia during CPB failed to demonstrate any difference in markers of organ damage, or length of stay in a prospective clinical study in nearly 300 patients.^17^ In the study by McGuinness et al., the target P_a_O_2_ of the intervention group was 75 to 90 mmHg, but this goal was only reached after more than 20 min, stressing the importance of advancing goal-directed perfusion technique to reach fast group separation in clinical studies that focus on the effects of oxygen therapy during CPB. In a study by Shaefi et al., it took nearly 45 min to separate the normoxia from the hyperoxia group,^28^ which may have been one of the reasons why the study did not find any difference between the groups in terms of neurocognition after surgery.

On a calculatory basis, we would have expected to reach the targeted P_a_O_2_ corridor more often by applying our formula. However, the formula is based on a couple of assumptions and pre-calculations, which retrospectively proved not to fit our patient cohort (Table 5). Firstly, BSA is a major factor in the determination of our mathematical deductions. As Redlarski et al. show, discrepancies between most of the known BSA formulae can reach 0.5  m^2^ for the standard adult physique.^29^ As in most medical cases, we used the DuBois formula, but it might be necessary to also include the age and sex of the patients, too. However, body variables used need to be easy-to-identify to make the formula applicable in practice. The same is true for the calculation of blood volume. Patient blood volume impacts most facets of perfusion technique.^30^ Blood volume in our study was calculated by a factor of 65 mL/kg/KG for women and 75 mL/kg/KG for men, but we observed meaningful differences between the pre-calculated values and the volume calculated with the knowledge after CPB initiation even when comparing formulas by both Mosteller and Nadler.^21^

Most importantly, the standard CI we preassumed (2,5 L/min/m^2^) only roughly fit in those patients with a resulting P_a_O_2_ between 150 and 249.9 mmHg. In the other patient cohorts, there were meaningful deviations. However, without more invasive procedures, there is no easily-available possibility to reach valid values for CI ahead of CPB, unless measured in a preliminary cardiac catheterization. We would recommend to always use this value whenever available instead of a fixed value of 2,5 L/min/m^2^. In follow-up studies, we suggest to gain further information on the patient’s individual needs by assessing cardiac output at rest, for instance, via transthoracic echocardiography in the days ahead of surgery. Although the method has its own limitations, this non-invasive method could at least approximate the individual CI. Using this as a calculatory basis would presumably reach better results when applying the formula.

Possibly, further patient-intrinsic hemodynamic and (patho-)physiological conditions and how these change under certain influences before and during the initiation of CPB need to be anticipated that were not included in the formula such as level of sedation and analgesics as well as changes in circulatory and ventilatory support. Further influencing factors such as fluctuations in cardiac function, blood loss, altered temperature and fluid shifts may have also influenced our results. Nevertheless, applying our formula, all of our patients reached a P_a_O_2_ that is considered “safe” in common practice. The lowest value was 61.3 mmHg. The lowest P_a_O_2_ not accompanied by any other signs of hypoxia such as an increase in lactate or central venous oxygen saturation <75%. Arterial hemoglobin oxygen saturation (S_a_O_2_) was always above 90%.

Presumably, the target range we used in our study may not be the optimum for every patient as the optimum arterial oxygen pressure probably very much depends on the clinical condition. As a result of the tempting assumption that supranormal arterial oxygen tension will improve oxygen delivery to peripheral tissue, very high values of PaO_2_ are frequently encountered in routine clinical practice. We also observed this in the pre-HLM-period, during which we measured a mean P_a_O_2_ of 214 ± 96 mmHg. However, the reason behind this may also be due to the fact that anesthetists often use this time to conduct tasks such as echocardiography or the surgeon may require apnea for instance during preparation of the internal mammary artery, which may necessitate this additional margin of safety.

As regards tissue oxygenation, a worrying observation is that all of our patients were anemic before the initiation of the HLM, although we already excluded those with a severe anemia of hemoglobin values below 9 g/dL. Studies show that even ultra-short-term anemia treatment can be effective in patients undergoing cardiac surgery, which helps to reduce allogeneic transfusions.^31^ As hemoglobin serves as the primary vehicle to transport oxygen to hypoxic tissue, using every chance to increase autologous hemoglobin should be a major focus in cardiac surgery, especially in those clinicians that seem to value hyperoxia as seen in the supranormal P_a_O_2_ in the pre-HLM period.

In conclusion, the targeted range of P_a_O_2_ was only reached in a third of our patients. However, none of our patients experienced severe hypo- or hyperoxia, so that the formula may not be unerring, but at least safe all the same and therefore a good starting point for future development. Despite the above mentioned limitations, our pilot study provides data that can be used as the basis for more definitive clinical trials – and we show here why that would be so important to do.
